# High-Power-Efficiency Readout Circuit Employing Average Capacitance-to-Voltage Converter for Micro-Electro-Mechanical System Capacitive Accelerometers

**DOI:** 10.3390/s23208547

**Published:** 2023-10-18

**Authors:** Linxi Li, Xinquan Lai, Yuheng Wang, Zhiwen Niu

**Affiliations:** 1 School of Electronic Engineering, Xidian University, Xi’an 710071, China; lilinxi4213@stu.xidian.edu.cn (L.L.); xqlai@mail.xidian.edu.cn (X.L.); zwniu@stu.xidian.edu.cn (Z.N.); 2 Shenzhen Changyuntong Semiconductor Co., Ltd., Shenzhen 518133, China; 3 College of Electronics Information, Qingdao University, Qingdao 266071, China

**Keywords:** readout circuit, capacitance-to-voltage converter, low noise, low power, MEMS accelerometer, capacitive, high power efficiency

## Abstract

A capacitance-to-voltage converter (CVC) is proposed in this paper and applied to a readout circuit for a micro-electro-mechanical system (MEMS) accelerometer to improve the power efficiency. In a traditional readout circuit, the front-end CVC has to operate at a high sampling frequency to resist thermal noise deterioration due to the large parasitic capacitance introduced by the mechanical sensing element. Thus, the back-end analog-to-digital converter (ADC) also has to operate at a high sampling frequency to avoid noise aliasing when sampling the output signal of the CVC, which leads to high power consumption. The average CVC technique is proposed in this paper to reduce the sampling frequency requirement of the back-end ADC and thus reduce the power consumption. Both the traditional readout circuit and the proposed readout circuit are simulated with a commercial 0.18 μm BCD process. The simulation results show that noise aliasing occurs, and the noise power spectral density (PSD) of the traditional readout circuit increases by 12 dB when the sampling frequency of back-end ADC is reduced by 24 dB. However, in the proposed readout circuit, a noise aliasing effect does not occur. Moreover, the proposed readout circuit reduces the power consumption by 53% without thermal noise deterioration. In addition, the proposed CVC circuits are fabricated in an 0.18 μm BCD process, and the test results show that the presented readout circuit based on the average CVC technique can obtain better performance than the traditional CVC-based readout circuit.

## 1. Introduction

The micro-electro-mechanical system (MEMS) is developed based on microelectronics technology, integrated circuit technology, and processing technology. It focusses on ultra precision mechanical processing, involving various disciplines such as microelectronics, materials, mechanics, and chemistry, and is widely used in MEMS accelerometers, MEMS optical sensors, MEMS pressure sensors, MEMS gyroscopes, and so on.

MEMS capacitive accelerometers play an important role in inertial measurement units [1,2,3], platform stabilization systems [4,5,6,7,8,9,10], structural health monitoring [11,12,13,14,15], and tilt sensing [16,17]. In these applications, the MEMS capacitive accelerometers are powered by batteries; thus, high power efficiency is required to extend the battery life. The key point to improving power efficiency is innovation and the optimization of the readout circuit. The readout circuit is, namely, the signal processing circuit, which is designed to measure the external acceleration by detecting the capacitance change of the mechanical part of the MEMS structure. In order to achieve low power consumption, the readout circuits of MEMS capacitive accelerometers can be designed with an open-loop architecture (e.g., charge control readout and voltage control readout), rather than a closed-loop one (e.g., force feedback readout) [18,19,20,21].

In the open-loop readout structure, one of the main issues is that the nonlinearity of the mechanical sensing element significantly increases with the increase in capacitance variation of the sensing element due to its reciprocal trans-function. Therefore, the input range of the sensing element is limited to a small level to suppress the sensing element’s nonlinearity [22]. As a result, the capacitance variation of the sensing element (femto-farad level) is much lower than the capacitance of the parasitic capacitance of the sensing element (pico-farad level), which results in a significant deterioration of gain error and thermal noise. The oversampling successive approximation (OSA) readout technique and the Nested-OSA technique were reported to deal with these deterioration problems [23,24]. However, a high sampling frequency of the capacitance-to-voltage converter (CVC) based on the OSA or Nested-OSA technique leads to high power consumption of the readout circuit [25,26]. The bandwidth-enhanced OSA readout technique can be employed for noise floor improvement, but an auxiliary circuit is needed to increase the response of the system, which reduces the power efficiency [27]. Although the reset noise sampling feedforward (RNSF) technique can deal with the parasitic-induced noise, it complicates the circuit and consumes too much chip area [28]. To resolve this shortcoming mentioned above, a novel average CVC method is proposed in this paper to reduce the power consumption of the readout circuit by reducing the sampling frequency requirement of the back-end circuits without deteriorating the noise floor of the MEMS capacitive accelerometer. The technique of the average CVC is averaging several sampled capacitance values in capacitive detection to reduce the operating frequency of the subsequent circuits.

The rest of the paper is organized as follows: Section 2 provides a performance analysis of the traditional readout circuit of the MEMS capacitive accelerometer. In Section 3, the principles and performance of the readout circuit employing the average CVC technique are described in detail. In Section 4 and Section 5, the simulation results and physical measurement results are presented. The conclusions are then drawn in Section 6.

## 2. Traditional Readout System of MEMS Capacitive Accelerometer

For the open-loop readout circuit with significant input parasitic capacitance, the overall noise performance of the MEMS capacitive accelerometer readout system and the trade-off between noise and power is determined by the front-end switched-capacitor capacitance-to-voltage converter (SC-CVC) and the back-end analog-to-digital converter (ADC). In this section, the sensor structure and the traditional readout circuit of the MEMS capacitive accelerometer are analyzed in detail.

### 2.1. Sensor Structure

**Figure 1:**
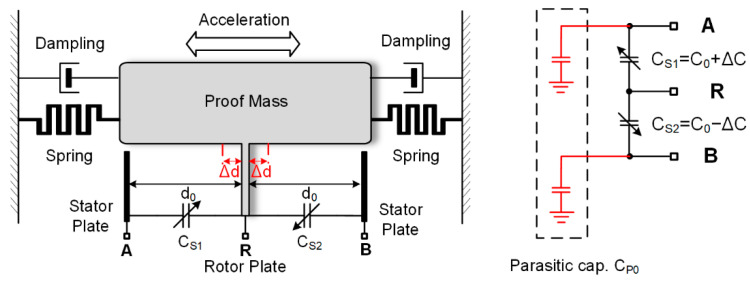
Sensing element of a typical MEMS capacitive accelerometer.

Figure 1 shows one sensing element in a typical open-loop MEMS capacitive accelerometer. The mechanical sensor consists of a moving proof mass suspended on springs over a substrate and a set of fixed electrodes. Applying an external acceleration to the MEMS system leads to the deflection of the proof mass from its center position, resulting in a differential capacitance change. The sensing capacitances $C_{S1}$ and $C_{S2}$ are the parallel-plate capacitors formed by the stator plates (unmovable plates connected to the sensing electrodes A and B) and the rotor plates (movable plates on the proof mass connected to the driving electrode R via spring), which are expressed below.

(1) $$C_{S1}=C_{0}\left(\frac{1}{1-∆d/d_{0}}\right)\approx C_{0}\left(1+∆d/d_{0}\right)=C_{0}+∆C_{S}\;C_{S2}=C_{0}\left(\frac{1}{1+∆d/d_{0}}\right)\approx C_{0}\left(1-∆d/d_{0}\right)=C_{0}-∆C_{S}$$

where $C_{0}$ is the rest capacitance of the sensing capacitor, $d_{0}$ is the rest distance between a pair of rotor plates and stator plates, which form the sensing capacitor, ∆d is the displacement, which is in linear proportion to the acceleration signal. Equation (1) shows that the relationship between the capacitance variation of the differential parallel-plate capacitor and the acceleration signal (displacement) is approximately linear.

### 2.2. Traditional Readout Circuit for MEMS Capacitive Accelerometer

According to Equation (1), in order to suppress the nonlinearity to an acceptable level, the capacitance has to be limited to several femto-farad levels for an acceptable die cost. However, in fact that the femto-farad-level capacitance variation in the sensing element is much lower than the parasitic capacitance of the sensing electrodes, which is at the pico-farad level, results in signal-to-noise (SNR) deterioration. In order to reduce the effect of the wideband parasitic-induced noise, the CVC should operate at a high frequency because the oversampling technique can effectively suppress the wideband noise.

**Figure 2:**
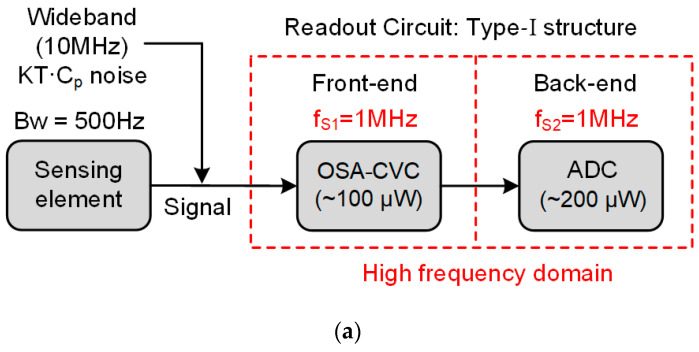

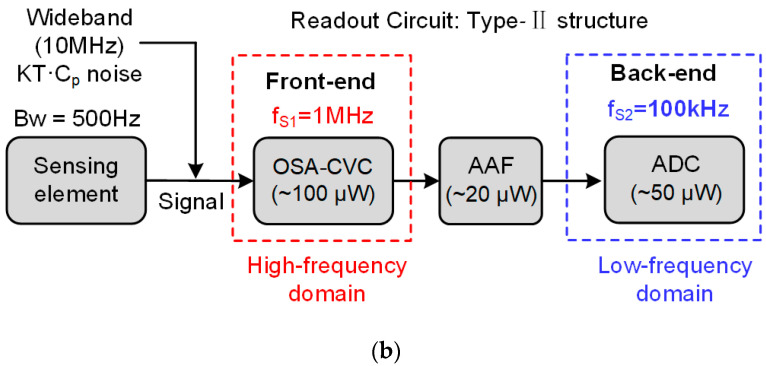
Traditional readout circuit for MEMS capacitive accelerometer (**a**) Type-Ⅰ structure (**b**) Type-Ⅱ structure.

The traditional readout circuit of the MEMS capacitive accelerometer is summarized as Type-Ⅰ and Type-Ⅱ structures, as shown in Figure 2. For both of them, the front end is referred to as the capacitance-to-voltage converter, and the back end is referred to as the ADC (usually the three-order sigma–delta ADC) based on the signal processing circuit. For the Type-Ⅰ structure, which is shown in the Figure 2a, the wideband noise caused by the parasitic capacitance will deteriorate the noise floor of the MEMS capacitive accelerometer. The CVC should operate in a high-frequency domain to reduce the SNR deterioration. The ADC is directly connected to the front-end OSA-CVC and shares the same sampling frequency as that of OSA-CVC. Thus means the ADC also operates in the high-frequency domain. The ADC uses a low sampling frequency to sample the CVC’s high bandwidth output ($f_{S1}/2=5$00 kHz), which will result in noise aliasing and noise PSD (power spectrum density) deterioration [29,30,31]. Furthermore, this structure will also lead to significant power waste as the bandwidth of signal from the MEMS accelerometer is far smaller (typical 500 Hz) than the sampling frequency of the CVC (typical 1 MHz). 

The Type-II structure, as shown in Figure 2b, employs an anti-alias filter (AAF) in the signal path. The AAF is inserted between the CVC and the ADC. It is used to isolate the CVC from the ADC. This means the ADC is not connected to the CVC directly. The high-frequency noise and harmonics are significantly suppressed by the AAF. Then, the back-end ADC can operate in the low-frequency domain. Compared to the Type-Ⅰ structure, the Type-Ⅱ structure consumes less power without sacrificing the resolution of the signal. However, the readout structure is more complex, and it consumes more chip area.

### 2.3. Capacitance to Voltage Converter

**Figure 3:**
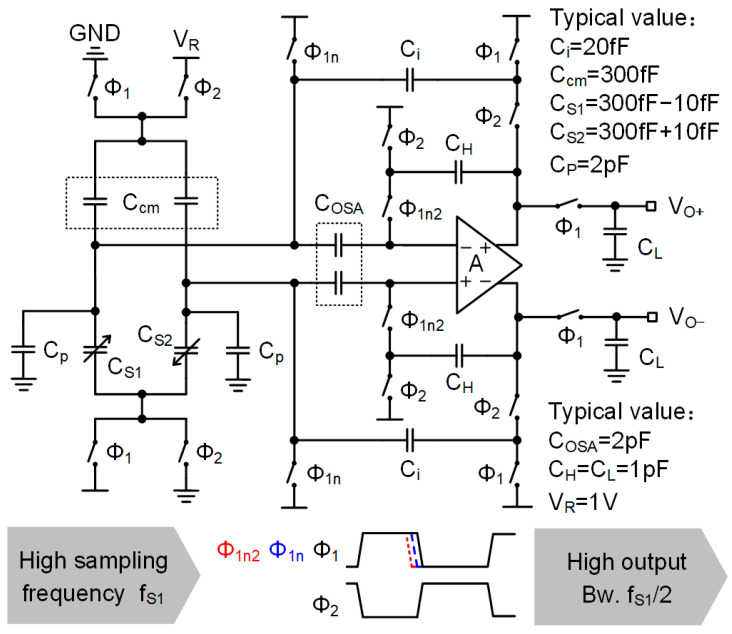
Schematic of front-end OSA-CVC for MEMS capacitive accelerometer.

A schematic of the SC-CVC based on the OSA (oversampling successive approximation) technique is shown in Figure 3 [24]. The OSA-CVC operates at a sampling frequency (1 MHz) much higher than that of the signal bandwidth (500 Hz) for two reasons. One reason is to reduce gain error deterioration, and the another is to reduce the output noise PSD. This will be analyzed in detail below.

To illustrate the first reason, let us show the relationship between the output voltage of the OSA-CVC and the gain, which is expressed as below [24].

(2) $$V_{out}\left(n+N\right)=\frac{C_{s1}-C_{s2}}{C_{i}}V_{R}\left[1-{\left(\frac{1}{1+\frac{A_{0}}{\sigma_{d}}}\right)}^{N+1}\right]$$

where $C_{s1}$ and $C_{s2}$ are equivalent capacitors of the differential capacitive sensor, $C_{i}$ is the integration capacitor, $\sigma_{d}=2C_{P}/C_{i}$ is deterioration factor, $A_{0}$ is the gain of the operational amplifier, n is sampling period, and N is sampling step. Equation (2) indicates that the OSA-CVC undergoes multiple sampling steps to reduce gain error. This leads to an increase in the sampling frequency.

To explain the second reason, the relationship between the output noise PSD of the OSA-CVC and the sampling frequency is presented below [26]:

(3) $${PSD}_{1}=V_{n}=\sqrt{\frac{2}{f_{s1}}\frac{kTC_{p}}{C_{i}^{2}}+\frac{2}{f_{s1}}\frac{kT\gamma \alpha C_{p}}{C_{L}C_{i}}}$$

where $f_{s1}$ is the sampling frequency, $C_{P}$ is the parasitic capacitance between the sensing electrodes and the ground, $C_{L}$ is the load capacitor, α is a constant depending on the structure of input stage of the amplifier with a typical value between 1.0 and 2.0, γ is a constant depending on the process, with a typical value of 0.6 [26]. The Equation (2) omits less important capacitances to simplify the expression. Equation (3) indicates that the noise PSD is deteriorated due to the parasitic capacitance, $C_{P},$ and, thus, a high sampling frequency $f_{s1}$ is required (typically 1 MHz) to deal with the wideband (typically 10 MHz) noise from parasitic capacitance, $C_{P}$.

## 3. Proposed Average CVC

**Figure 4:**
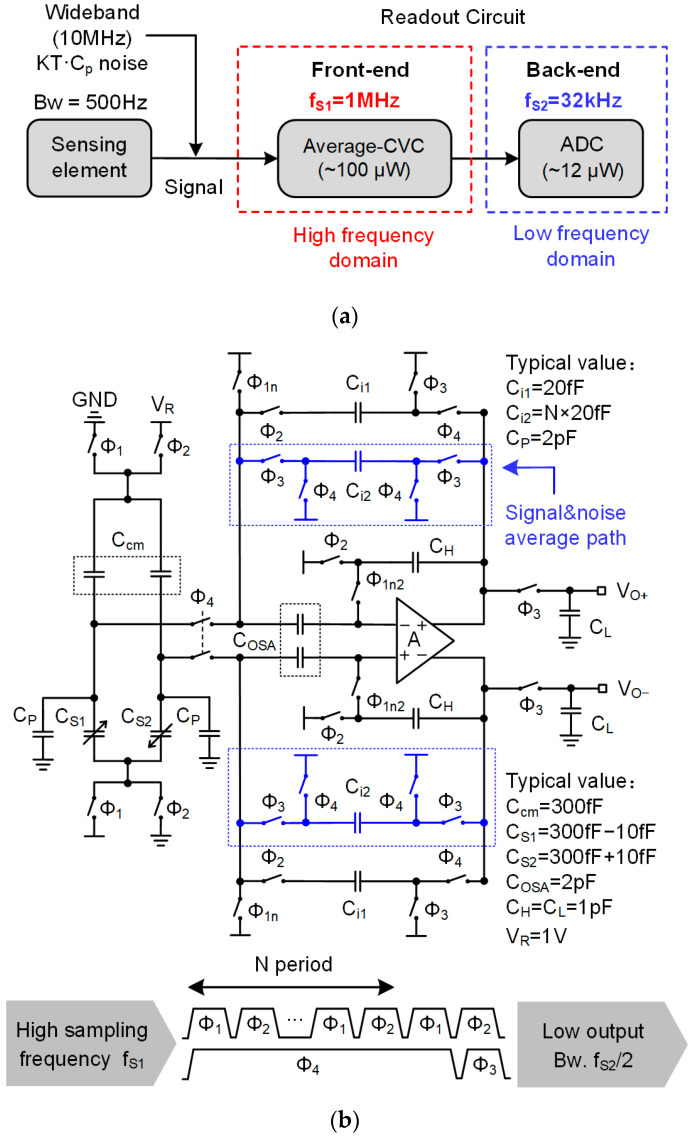
Proposed readout circuit for MEMS capacitive accelerometer (**a**) Diagram of system (**b**) Schematic of front-end average CVC.

Thus, in the proposed readout shown in Figure 4a, the sampling frequency, $f_{S2},$ of the ADC is reduced to improve the power efficiency, while the CVC’s sampling frequency, $f_{S1},$ is kept constant to deal with the wideband noise from the parasitic capacitance, $C_{P}$. The reduction in $f_{S2}$ leads to two noise problems, which are addressed as follows.

The first problem is the increased quantization noise of the ADC. When the ADC’s sampling frequency, $f_{S2},$ is reduced while the signal bandwidth does not change, the oversampling ratio is reduced, and, thus, the quantization noise power is increased [27,28,29,30]. Thus, the lowest sampling frequency, $f_{S2},$ should guarantee that the quantization noise power does not dominate the CVC’s noise power so that the quantization noise can be ignored. In this work, the lowest sampling frequency, $f_{S2},$ is approximately 32 kHz. A simple calculation is helpful to explain it. According to reference [4], a three-order sigma–delta ADC with a one-bit quantizer can reach a peak SNR of 75 dB with a 1 V input signal level and an oversampling ratio of 32 (i.e., the signal bandwidth is 500 Hz, the while sampling frequency, $f_{S2},$ is 32 kHz). For the OSA-CVC, given the typical component values shown in Figure 2, the signal level is 1 V according to Equation (2), and the noise PSD is 7.3 μV/√Hz according to Equation (3). This is equivalent to an SNR of 60 dB within ADC’s bandwidth ($f_{S2}$/2). Thus, the SNR of the ADC (75 dB) is still much higher than that of the CVC (60 dB) at a 32 kHz sampling frequency. This means that ADC’s quantization noise can be ignored in the readout circuit given the condition that $f_{S2}$ is higher than 32 kHz.

The second problem is the increase in thermal noise due to noise aliasing. When the ADC uses a low sampling frequency ($f_{S2}$ = 32 kHz) to sample the CVC’s high bandwidth output ($f_{S1}/2\;$ = 500 kHz), noise aliasing will occur, and the noise PSD will deteriorate [31]. An average CVC is proposed to deal with this noise aliasing problem. As shown in Figure 4, the feature of the average CVC is that it is able to input a signal at a high sampling frequency ($f_{S1}$) as well as an output signal at a low bandwidth ($f_{S2}/2$) without noise aliasing. The principle of averaging the CVC is that the signal is a coherent source, and the averaging method does not change the signal power. The random noise can ideally be suppressed by averaging the repeated measured output values because the aliased thermal noise is a non-coherent noise that can be reduced by signal averaging [32,33,34,35].

The circuit implementation of the average CVC is shown in Figure 4b. The average CVC introduces a new $C_{i2}$ branch and new clock phases, Φ3 and Φ4, to carry out the signal averaging operation. The average CVC firstly samples the charge from the sensor to the capacitor $C_{i1}$ at a high sampling frequency, $f_{S1}$. Then, after N sampling steps, the signal charge in $C_{i1}$ is transferred to the capacitor $C_{i2}$ to be averaged ($C_{i2}=NC_{i1}$) and outputted. As a result, the output signal bandwidth is reduced by N times, and the noise aliasing effect is suppressed by the averaging operation. The output signal of the average CVC is

(4) $$V_{out}=N\frac{C_{s1}-C_{s2}}{C_{i1}}\times \frac{C_{i1}}{C_{i2}}=\frac{C_{s1}-C_{s2}}{C_{i1}},\;C_{i2}=NC_{i1}$$

The output signal power of the average CVC is

(5) $$V_{s,out}=\sqrt{{\left(\frac{V_{s-1}+V_{s-2}\ldots +V_{s-n}}{N}\right)}^{2}}=V_{s},\;{V_{s}=V}_{s-1}=V_{s-2}=\ldots =V_{s-n}$$

where $V_{s}$ represents the signal power of the original OSA-CVC. $V_{s-1}$ represents the signal power of the first sampling period, and so on, and $V_{s-n}$ represents the signal power of the *N*th sampling period. Equation (5) shows that the output signal power of the average CVC is equal to that of the original OSA-CVC. The signal power is not changed by the averaging operation. 

The output noise power of average CVC is

(6) $$V_{n,out}=\frac{\sqrt{\left({V_{n-1}}^{2}+{V_{n-2}}^{2}\ldots +{V_{n-n}}^{2}\right)}}{N}=\frac{V_{n}}{\sqrt{N}},\;{V_{n}=V}_{n-1}=V_{n-2}=\ldots =V_{n-n}$$

where $V_{n}$ represents the noise power of the original- CVC. $V_{n-1}$ represents the noise power of the first sampling period, and so on, and $V_{n-n}$ represents the noise power of the *N*th sampling period. Equation (6) shows that the noise power of the average CVC is reduced by the averaging operation.

As a result, the *SNR* of the average CVC is improved by

(7) $$∆SNR=\frac{V_{s,out}/V_{n,out}}{V_{s}/V_{n}}=\sqrt{N}$$

Compared with the original OSA-CVC shown in Figure 2, Equations (4) and (5) show that the output signal and signal power of the average CVC do not change. However, the noise power is reduced by $1/\sqrt{N},$ as expressed by Equation (6); therefore, the *SNR* is improved by a factor of $\sqrt{N}$.

## 4. Simulation Result

Both the traditional readout circuit employing the OSA-CVC (reproduced from reference [4]) and the proposed readout circuit employing the average CVC are simulated at the transistor level. The complete readout circuit includes five parts: (1) a front-end CVC, (2) a back-end ADC, (3) voltage and current references, (4) clock generators, and (5) output buffers.

The typical values of the important components of the front-end CVC are shown in Figure 2. The back-end ADC is a three-order discrete-time sigma–delta ADC with a one-bit quantizer. The structure of the ADC is a cascade of integrators with feedback (CIFB). The amplifiers used in the ADC have a 90 dB gain, 10 MHz unity-gain-bandwidth at a 2 pF capacitive load, 10 nV/√Hz input noise, and 40 μA/20 μA/5 μA/1.3 μA current consumption for a 1 MHz/500 kHz/125 kHz/31.25 kHz sampling frequency with a 1.8 V supply voltage.

**Figure 5:**
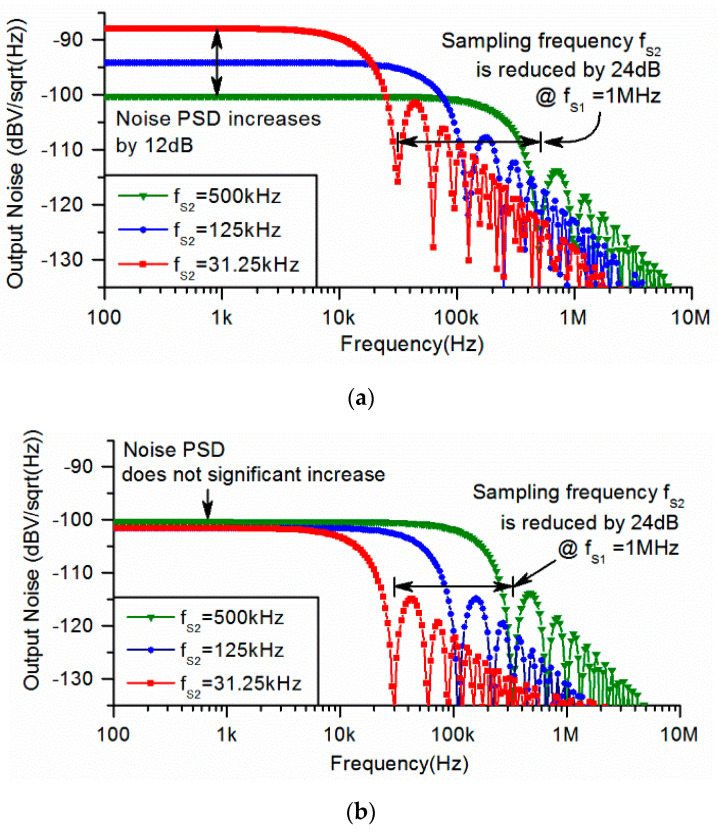
PSDs of readout circuit with reduced sampling frequency. (**a**) Traditional readout circuit employing OSA-CVC. (**b**) Proposed readout circuit employing average CVC.

The noise PSDs of the readout circuit employing an OSA-CVC with different sampling frequencies, $f_{S2},$ are shown in Figure 5a. Given the sampling frequency $f_{S1}$ of the CVC being constant at 1 MHz, when the sampling frequency $f_{S2}$ of the ADC is reduced from 500 kHz to 31.25 kHz, the power consumption of the whole readout circuit is reduced by 56%, from 250 μW to 110 μW. However, the noise PSD increases by 12 dB, from −100 dBV to −88 dBV. The noise PSDs of the proposed readout circuit are shown in Figure 5b. Given $f_{s1}$ being constant at 1 MHz, when $f_{S2}$ is reduced from 500 kHz to 31.25 kHz, the power consumption of the readout circuit is reduced by 54% from 255 μW to 115 μW. However, the noise PSD is not significantly increased. A comparison of these two readout circuits is shown in Table 1. It should be noticed that the proposed readout circuit can also adapt to different types of MEMS accelerometers by adjusting the number and value of integrated capacitors and average cycle numbers. Therefore, the size and the complexity of the MEMS accelerometer are relatively less important and less discussed in this paper.

## 5. Experimental Result

**Figure 6:**
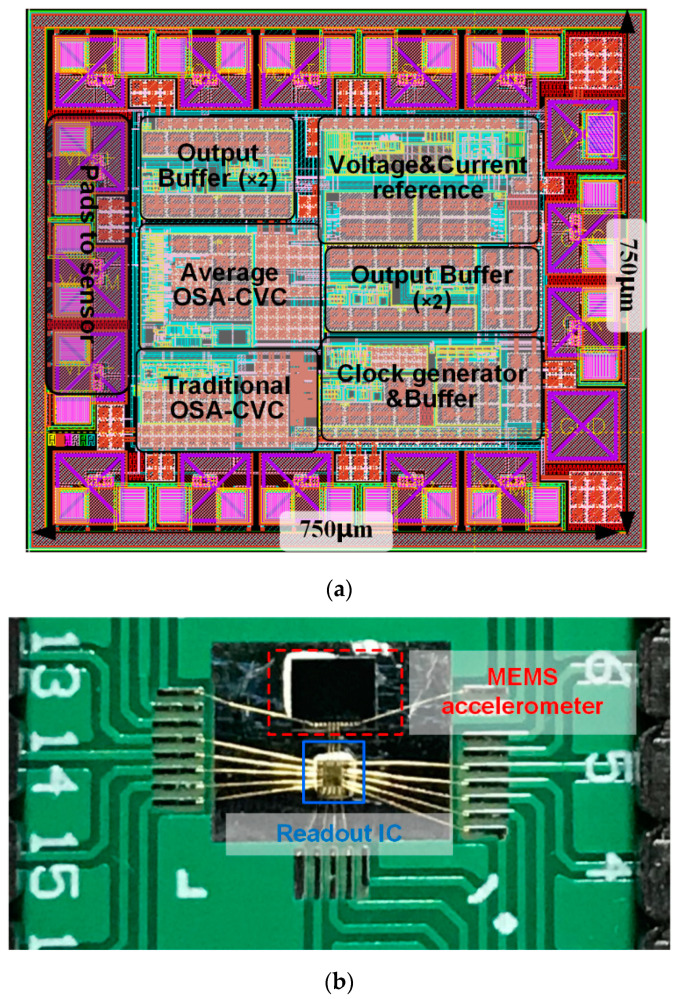
Photograph of the readout circuit tested with a MEMS accelerometer. (**a**) Layout of design. (**b**) Test photo.

**Figure 7:**
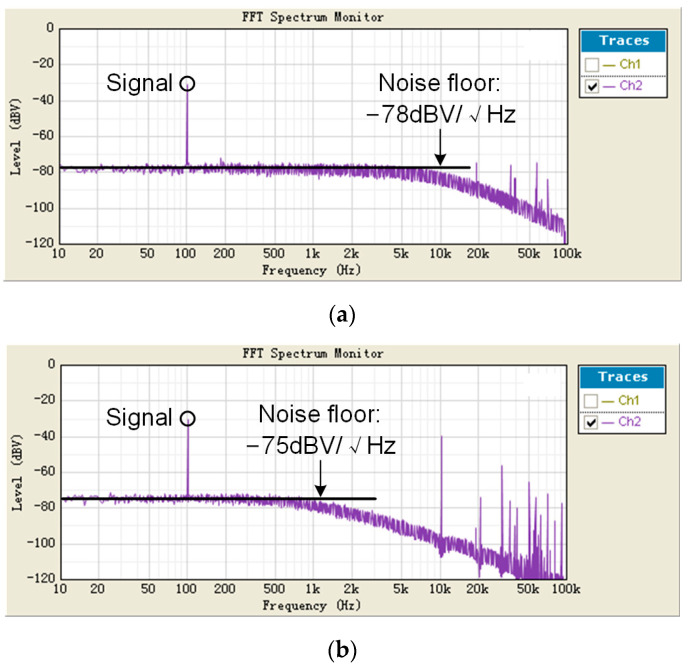
Measurement PSDs of the readout circuit. (**a**) Traditional readout circuit with 100 kHz sampling frequency. (**b**) Proposed readout circuit with 10 kHz sampling frequency.

The proposed CVC is demonstrated in a readout integrated circuit (IC) fabricated in a 0.18 μm BCD process. The traditional CVC (shown in Figure 1) is also placed in the same IC in order to give a comparison. The readout IC is tested with a commercial IoT MEMS accelerometer. Only the *X*-axis sensing element is used in this test to demonstrate the proposed readout circuit, as the sensing elements for other axes are similar. The photographs are shown in Figure 6. The measurement results show that when using the traditional CVC to readout the accelerometer, the output noise floor is −80 dBV/√Hz with a 100 kHz sampling frequency, as shown in Figure 7a, and when using the proposed CVC to readout the accelerometer, the output noise floor is −75 dBV/√Hz with a 10 kHz sampling frequency. Thus, with the proposed average CVC, the sampling frequency of readout circuit is reduced by 20 dB, and, thus, the power consumption is reduced by 20 dB, while the noise floor does not significantly increase (by only 3 dB).

The main experimental parameters of this work are listed in Table 2. Generally, the figure of merit (FoM) is used to evaluate the power efficiency of the MEMS accelerometer,

(8) $$FoM\;[W\cdot F/Hz]=\frac{Power\times Noise\;floor}{\sqrt{Bw}}$$

As can be seen from the table, this work has achieved a better *FoM* compared to others, apart from Refs. [7,28]. Ref. [7] benefits from a static high-voltage bias but sacrifices the ability to detect the DC signal. Ref. [28] consumes too much chip area compared to the proposed technique.

## 6. Conclusions

This paper proposed a power-efficient readout circuit for an MEMS capacitive accelerometer. Gain error deterioration and thermal noise deterioration are typical problems in MEMS accelerometers, which can be alleviated by a traditional OSA readout circuit. However, the power consumption of the traditional OSA readout circuit is high due to the high sampling frequency. Thus, the back-end analog-to-digital converter (ADC) also has to operate at a high sampling frequency to avoid noise aliasing when sampling the output signal of the CVC, which leads to high power consumption. The average CVC is proposed in this paper to reduce the sampling frequency requirement of the back-end ADC and thus reduce the power consumption without deteriorating the noise performance of the system. The experimental results show that, compared to the traditional readout circuit, the proposed readout circuit employing an average CVC can significantly reduce the power consumption of the readout circuit (by 20 dB), while the noise PSD does not significantly increase (by only 3 dB). It should be noted that although this proposed technique spends extra time establishing the signal and average noise power, it still has some practical value for the MEMS accelerometer when the requirement for system speed is not particularly high, considering its outstanding power efficiency.

## Figures and Tables

**Table 1:** Performance comparison table.

Reference/Paper	Ref [4] Reproduced in This Paper		This Work
Power/supply	250 μW/1.8 V	110 μW/1.8 V	115 μW/1.8 V
Sampling frequency	CVC: 1 MHz ADC: 1 MHZ	CVC: 1 MHz ADC: 31.25 kHz	CVC: 1 MHz ADC: 31.25 kHz
Output noise	−100 dBV/√Hz	−88 dBV/√Hz	−100 dBV/√Hz

**Table 2:** Comparison of readout circuit for open-loop MEMS capacitive sensor.

Reference/Paper	Zhong [27]	Yang [7]	Wang [28]	Yucetas [11]	This Work
Technique/Structure	Bandwidth-Enhanced OSA	High Voltage-Bias	RNSF	Traditional Oversampling	Averaged CVC
Full scale (g)	±8	±1.5	±8	±1.15	±4
Bandwidth (Hz)	10 k	200	1 k	200	1 k
Sampling rate (Hz)	100 k	-	50 k	-	10 k
Sensor sens (fF/g)	1.0	-	4	-	4
IC sens (mV/fF)	90	-	−100	-	90
$\mathrm{Noise}\;\mathrm{floor}\;(\mu g/\sqrt{Hz}$)	900	121	250	2.0	320
Power (μW)	248	0.184	62	3600	40
Supply voltage (V)	1.8	-	1.8	3.6	1.8
FOM (μW·μg/Hz)	2232	1.59	490	509	404
